# Green synthesis of Mn-doped iron oxide nanoparticles using sugarcane juice for magnetic hyperthermia applications

**DOI:** 10.1038/s41598-025-11998-5

**Published:** 2025-08-06

**Authors:** A. A. Hosny, Bahig El-Deeb, Z. A. Mohamed, Abdelwahab Hassan, Mohamed Kamel, Shehab Elbeltagi, H. A. A. Saadallah, E.M.M Ibrahim

**Affiliations:** 1 Physics Department, Faculty of Science, Sohag University, Sohag, 82524 Egypt; 2 Botany & Microbiology Department, Faculty of Science, Sohag University, Sohag, 82524 Egypt; 3 Science Department, Rustaq College of Education, University of Technology and Applied Sciences, PO Box 10, Al Rustaq 329, Sultanate of Oman; 4 Department of Physics, Faculty of Science, Fayoum University, Fayoum, 63514 Egypt; 5 Department of Physics, Faculty of Science, New Valley University, New Valley, 72511 Egypt

**Keywords:** Iron oxide, Superparamagnetic, Hyperthermia, Green synthesis, Sugarcane, Nanoparticles, Biophysics, Materials science, Nanoscience and technology, Physics

## Abstract

In this study, ecofriendly un-doped (0%), 5%, and 9% Mn-doped iron oxide nanoparticles were synthesized using sugarcane juice as reducing agents. X-ray diffraction (XRD) confirmed the high purity and crystalline nature of the undoped and Mn-doped iron oxide nanoparticles. Fourier transform infrared spectroscopy (FTIR) was used to investigate the surface functional groups involved during the reducing and capping process. The internal structure of the particles was examined using high resolution transmission electron microscope (HRTEM). The particles exhibited semi-spherical shapes, with mean particle size of 9.3, 9.5 and 13.5 nm for the 0, 5 and 9% Mn-doped samples, respectively. The magnetic properties of the nanoparticles were measured by studying the magnetic field dependence of magnetization at 300 K and 2 K up to 4 T. The samples displayed hysteretic behavior characteristic of ferromagnetic materials at 2 K and typical superparamagnetic features at 300 K. The magnetic heating properties under AC magnetic fields were investigated to assess the feasibility of the synthesized NPs for magnetic hyperthermia application. The specific absorption rate (SAR) values of the iron oxide nanoparticles increased with the Mn-doping level. The results suggest that the green synthesis of un-doped and Mn-doped iron oxide nanoparticles holds promising potential for magnetic hyperthermia treatment.

## Introduction

The biosynthesis routs using plant extracts is widely employed for the synthesis of metal and metal oxide nanoparticles (NPs), offering several advantages such as the generation of non-toxic byproducts, cost-effectiveness, ease of scaling up for large-scale production and enhanced biocompatibility, particularly in medical applications. Plants are rich in a variety of effective phytochemical compounds such as aldehydes, terpenoids, flavones, ketones, amides, phenols, ascorbic acids, sugars and carboxylic acids^1–3^.These constituents are capable of reducing metal salts into metal NPs with high stability even at large-scale production. Furthermore, the biomolecules present in plant not only reduce metal salts but also serve as capping agents, limiting nanoparticle growth and preventing agglomeration into large entities^4–6^. Various factors including metal salt concentration, plant extract concentration, pH, reaction time, and ambient temperature play crucial roles in tuning the purity, morphology, and particle size of employed biosynthesized NPs^7^. Biosynthesis has been successfully employed to produce various metals and metal oxides nanostructures (NSs) including Pt, Ag, Pd, Au, Fe_3_O_4_, TiO_2_, ZnO and, and, CuO^8–15^.

Iron oxide nanoparticles (NPs) represent a rapidly-growing area of research due to their excellent magnetic properties and biocompatibility. These features enable their broad applications in biomedical fields, including magnetic hyperthermia, magnetic resonance imaging, targeted drug delivery and biological magnetic separation, etc. Magnetic hyperthermia involves the generation of heat by magnetic NPs through applying AC magnetic field^16^. The phenomenon can be employed for cancer therapy by elevating the temperature at the tumor site above 42 °C which damages cancer cells and increases their sensitivity to radiation and anticancer drugs^17^. The heat generated by the NPs during each cycle of an applied magnetic field is directly related to the area of the hysteresis loop. Consequently, intrinsic parameters such as saturation magnetization and anisotropy of the NPs are highly effective for determining the hysteretic behavior^18^. The volume of the NPs is a key factor which determines their magnetization behavior and magnetization reversal mode^19^. Furthermore, the frequency and amplitude of the applied field as well as the temperature act as effective external parameters^20,21^. All these parameters significantly affect the relaxation mode of the magnetization of NPs which in turn affects the hysteresis behavior to the point of causing a complete disappearance of the coercivity (superparamagnetism) when the thermal energy and anisotropy energy barrier for magnetization reversal become comparable^22^. Producing high-quality superparamagnetic nanoparticles is essential due to their distinctive magnetic properties, such as large magnetic moments, high field irreversibility, and specific contributions from magnetic anisotropy. These characteristics are especially important in medical applications where preventing nanoparticle aggregation is critical. Achieving superparamagnetic behavior requires synthesizing ferromagnetic nanoparticles with sizes smaller than a specific critical threshold, typically below the magnetic domain size^23–25^.

Generally, the magnetic properties of iron oxide nanoparticles (IONPs) can be significantly improved by doping with a transition metal element, such as Ni, Co, Mg, Zn, Mn and etc. Fe_3_O_4_ has inverse spinel structure where half of Fe^3+^ ions occupy the tetrahedral A site, and all Fe^2+^ ions occupy the octahedral B site together with the other half of Fe^3+^ ions. Only the magnetic moment of the Fe^2+^ cation (4 *µ*B) contributes to the net magnetic moment of Fe_3_O_4_ because the magnetic moment of Fe^3+^ ions in A and B sites is compensated due to the antiferromagnetic coupling. Thus, substitution of Fe^2+^ cations by Mn^2+^ (magnetic moment = 5 *µ*B) can improve the net magnetic moments of Fe_3_O_4_^26^ which is crucial to enhance the heating efficiency. However, mixed valence of + 2 and + 3 of Mn cations makes the magnetic properties of Mn-doped Fe_3_O_4_ complex if compared to Zn-, Co- or Ni-doped Fe_3_O_4_^27^^28^, where many research groups reported that M_s_ of Fe_3_O_4_ NPs doesn’t vary monotonically with increasing the Mn doping level^29^. This has been attributed to that the magnetization depends not only on both the net magnetic moment of cations but also on the superexchange interaction between cations occupying the A and B sites.

Giri et al. synthesized ~ 10–12 nm Fe_1−x_Mn_x_Fe_2_O_4_ NPs (x = 0 to 1) using the co-precipitation method. Study of the magnetic properties and heating efficiency by calorimetric measurements (in a field of H_max_= 10–45 kA m^−1^ with frequency f_m_ = 300 kHz) showed that the maximum values of specific absorption rate SAR = 30 W/g and saturation magnetization M_s_ = 85 emu/g were recorded for x = 0.4 sample^30^. Jang et al. synthesized 15 nm-sized (Zn_x_Mn_1−x_)Fe_2_O_4_ NPs using a one-pot thermal decomposition method. The highest M_s_=175 emu/g and SAR = 432 W/g (at f_m_=500 kHz and H_max_ = 3.7 kA m − 1) were recorded for the x = 0.4 sample^31^. Recently, Yu et al. reported that increasing the Mn doping level from x = 0 to 0.7 in the 10 nm Mn_x_Fe_3.x_O_4_ decreases both the M_s_ value and heating efficiency due to the lattice distortion that weakened the superexchange interaction between B-site cations^32^.

The main aim of this work is to propose an effective and approach to synthesize Mn-doped iron oxide NPs using plant extracts from Sugarcane (*Saccharum officinarum*) as natural reducing and capping agents. Sugarcane juice (ScJ) mainly contains 70–75% water, 13–15% sucrose, and 10–15% fibers. It also contains nutritional supplements, including citric acid, malic acid, oxalic acid, and α-gluconic acid. In addition, the presence of various higher terpenoids, alcohol, fatty acids, flavonoids, glycosides, phytosterols, and phenolic acids have also been revealed^33,34^. So, it is an optimum choice to be used as natural synthesizer for NPs. Noteworthy, few studies have been reported for using ScJ as reducing agent in the green synthesis of different materials in nanosized scale. The synthesized NPs underwent a detailed investigation to assess their structural, morphological, and magnetic properties. Furthermore, the heating efficiency in AC magnetic field was examined and the specific absorption rate was determined to test the feasibility of their use as magnetic hyperthermia agents. This work contributes to the green chemistry field by demonstrating the potential of sugarcane juice in the fabrication of Mn-doped iron oxide NPs for biomedical applications.

## Results and discussion

### Structural studies

**Fig. 1:**
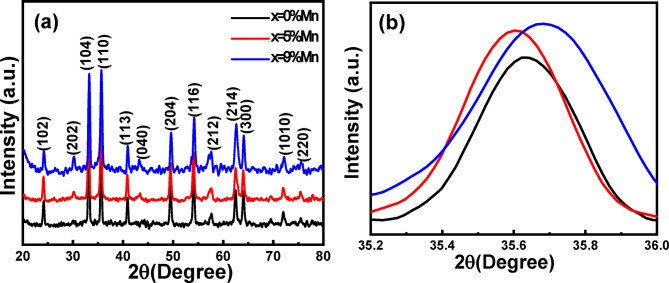
(**a**) The XRD patterns of the biosynthesized 0, 5, 9% Mn-doped iron oxide NPs. (**b**) the most intense peak (at 2θ= 35.6^°^ and crysllagroghic plane (110)) at expanded scale for all samples.

The sugarcane juice (ScJ)-mediated synthesis of pure and Mn-doped iron oxide nanoparticles (IONPs) with Mn concentrations of 0%, 5%, and 9% was confirmed by x-ray diffraction (XRD) analysis (Fig. 1a). The observed diffraction peaks (2θ^°^ (hkl)) 24.1° (102), 30.2° (202), 33.2° (104), 35.5° (110), 40.8° (113), 43.1° (040), 49.5° (204), 54.1° (116), 57.5° (212), 62.5° (214), 64° (300), 72.1° (1010),and 75.5°(220) correspond well to the Fe_3_O_4_ cubic spinel structure (JCPDS card number 01-088- 0315) and/or hematite Fe_2_O_3_ hexagonal structure (JCPDS card number 00-024-0072). It is important to note that differentiating between various iron oxide phases using XRD is challenging due to their very similar d-spacing values^35,36^.

The intensity of the Fe_3_O_4_ phase significantly increases with increasing the Mn doping levels indicating that Mn incorporation promotes the formation of Fe_3_O_4_ structure while suppressing the Fe_2_O_3_ phase. Similar results have been reported by Gupta et al.^37^ where the hematite peaks disappeared completely at x = 0.05 in the Mn_x_Fe_3−x_O_4_ system. Furthermore, the absence of any Mn-based secondary phases in the XRD patterns supports the successful incorporation of Mn^2+^ ions into the IONP lattice. Figure 1b shows the XRD patterns on an expanded scale, highlighting a slight shift in the most intense peak at the (110) plane to lower and higher theta for x = 5% and 9% Mn-doped samples, respectively, compared to the un-doped sample. This shift may be attributed to the presence of multiple oxidation states of both Fe and Mn, which leads to either expansion or contraction of the iron oxide lattice^38^. The average crystallite size *D*_*XRD*_ was calculated using the Debye–Sherrer’s Eq. (5 - Sect. 3.3.1). A significant decrease in *D*_*XRD*_ (Table 1) can be attributed to the structural distortion caused by Mn doping, as Mn^2+^ ions (0.92 Å) have a larger ionic radius than Fe^3+^ (0.64 Å) and Fe^2+^ (0.76 Å). Consequently, the lattice parameters and unit cell volume shrink (Table 1). Additionally, the internal local strain and dislocation density which indicate the degree of crystallographic defects in the samples were estimated using Eqs. (8 - Sect. 3.3.1) and (9 - Sect. 3.3.1), respectively. The data presented in Table (1) suggest that both internal strain and dislocation density increase with higher Mn doping levels. This indicates that the incorporation of Mn²⁺ ions into the iron oxide lattice results in more crystalline defects.

**Table 1:** Lattice parameter (*a*,* c*), cell volume (*V*), crystallite size *D*_*XRD*_, microstrain *η*, and dislocation density *δ* for pure and Mn-doped ionps.

Mn content	a(Å)	c(Å)	V(Å^3^)	D_XRD_ (nm)	Micro strain η ˣ 10^−4^	Dislocation density δˣ10^15^ (Line/m^2^)
0%	5.033	13.76	301.9	16.3	21.26	3.76
5%	5.032	13.73	301.2	15.93	21.75	3.93
9%	5.025	13.71	299.9	15.92	21.77	3.94

**Fig. 2:**
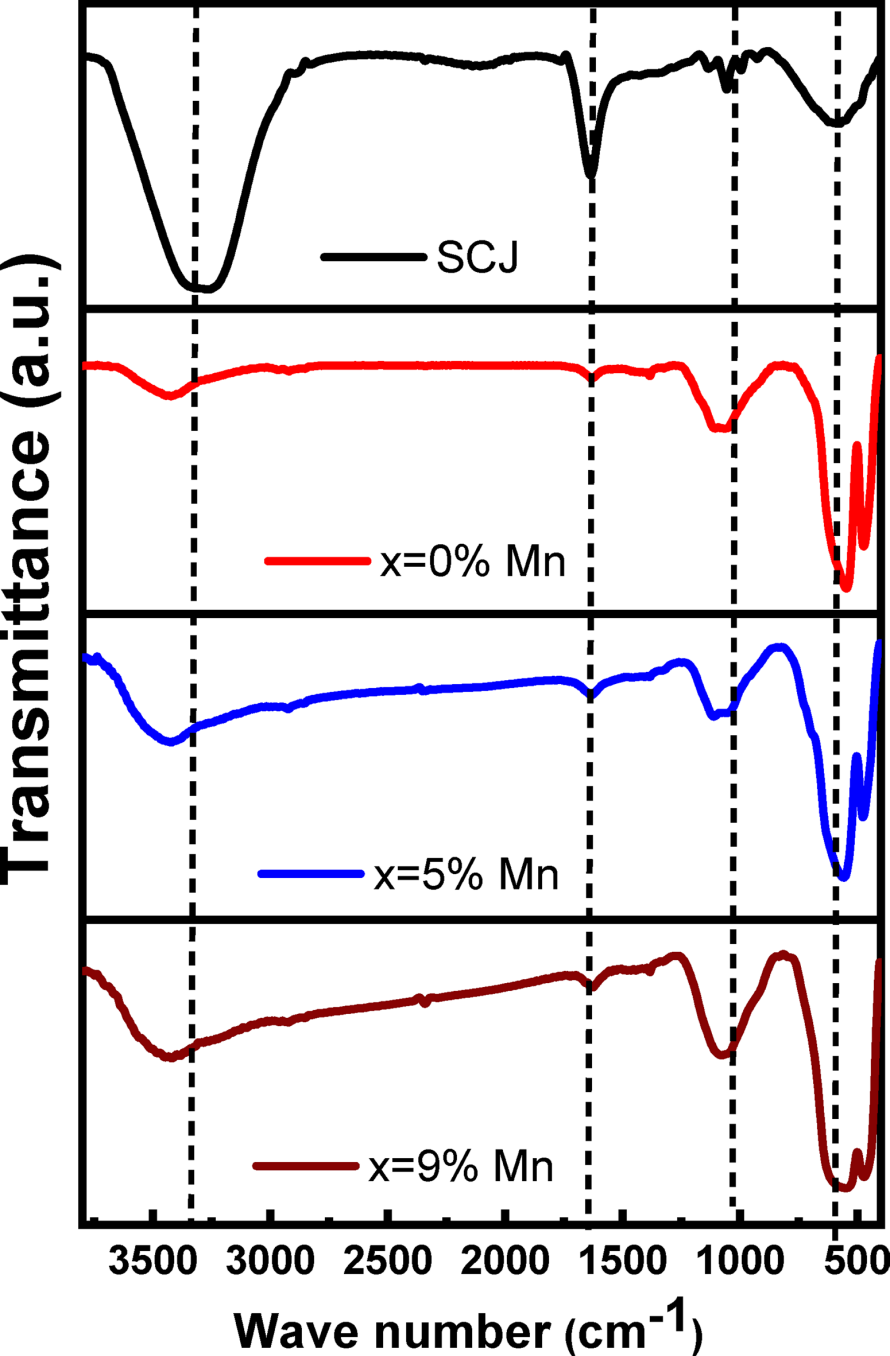
FTIR spectrum for ScJ, 0, 7, 9% Mn-doped iron oxide.

The functional chemical groups present in ScJ (control) and the resulting iron oxide nanoparticles (IONPs) were analyzed using Fourier-transform infrared (FTIR) spectroscopy in the range of 400–4000 cm^−1^ (Fig. 2). Four characteristic peaks were observed in the ScJ spectrum at approximately 3321, 1645, 1060, and 597 cm^−1^ corresponding to hydroxyl group (O-H, C = O stretch group, O-C = O, and bending vibration = C–H, respectively^39,40^. On the other hand, there are similar peaks in biosynthesized samples in comparison to control spectrum (ScJ) but with different intensity and shift to different position of wave number. The observed shift and intensity variation of the main absorbance bands for pure and Mn-doped iron oxides refer to that the biomolecules in ScJ are responsible of reduction and biosynthesis of iron oxide NPs^11^. In addition, the peaks from 400 to 500 cm^−1^ correspond to the stretching vibration mode of Fe–O and Mn-O^39^. FTIR results suggest presence of sucrose, fructose, and other biomolecules namely, amino acids and organic acids on the surface of resultant NPs which work as capping agents^40–42^.

### Morphological study

**Fig. 3:**
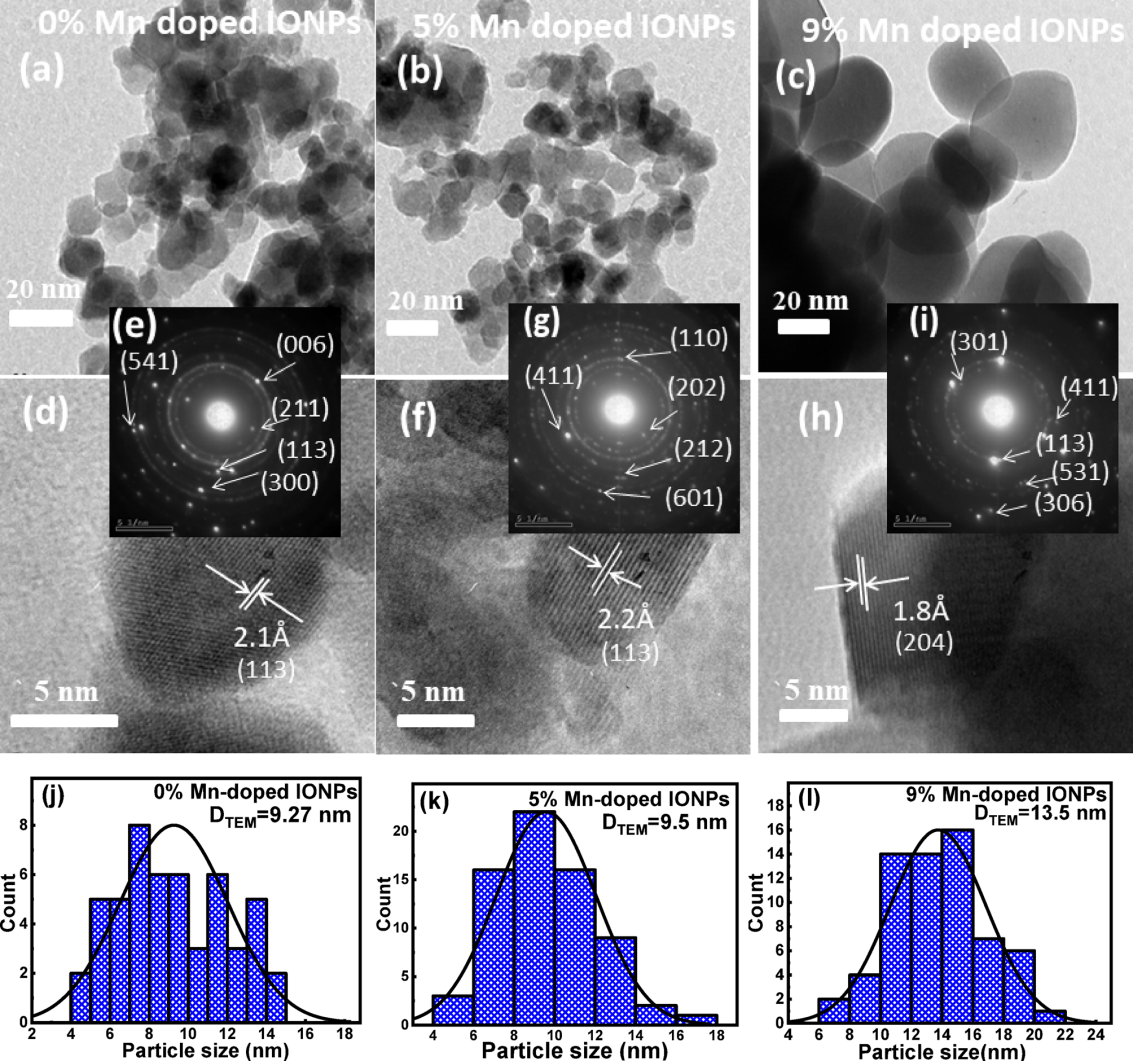
(**a**, **b** & **c**) HRTEM images, (**d**, **f** & **h**) HRTEM at higher magnifications, (**e**, **g** & **i**) SAED patterns and **j**, **k** & **l**) Histogram representation of particle size distribution of 0%, 5% and 9% Mn-doped IONPs, respectively.

High-resolution transmission electron microscopy (HRTEM) was used to gain insight into the internal structure of the synthesized nanomaterials. Figure 3 illustrates the HRTEM results for the 0%, 5%, and 9% Mn doped-samples.

The images depicted in Fig. 3 (a-c) confirm that the samples consist of semi-spherical particles at the nanoscale. Average particle size of 9.27 nm, 9.5 nm, and 13.5 nm for the 0%, 5%, and 9% Mn-doped samples, respectively was estimated from the corresponding range of histogram with Gauss curve fitting and its distribution depicted in Fig. 3j, k &l). Mn atoms at grain boundaries promote mass transport and grain coalescence, resulting in slight increase in the size of the particles by Mn doping^43^. The atomic structure of the iron oxide nanoparticles (IONPs) was further analyzed using HRTEM images at higher magnifications (Fig. 3d, f and h). The images reveal distinct lattice fringes (denoted by short white parallel lines) indicating the high crystallinity of the synthesized nanoparticles (NPs). However, each IONP appears to be surrounded by a thin poorly crystalline shell which significantly influences the magnetic properties of the samples as will be discussed latter. The selected area electron diffraction (SAED) patterns of the synthesized NPs are illustrated in Fig. 3e, g& i. The patterns confirm the good crystallinity of the NPs and coincide with results of the XRD investigation.

### Magnetic properties

**Fig. 4:**
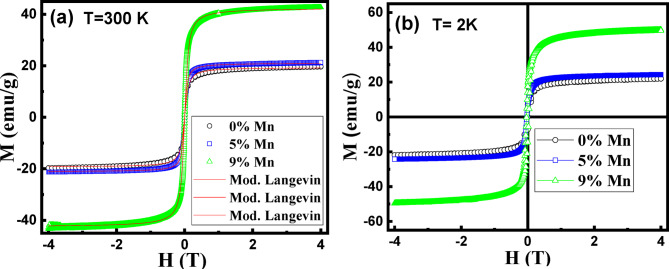
Hysteresis loops at (**a**) 300 K and (**b**) 2 K for 0, 5 and 9% Mn-doped IONPs.

The magnetic properties of the synthesized samples were studied by measuring the magnetic field (H) dependency of the magnetization (M) at 300 and 2 K and temperature (T) dependency of M at a constant external applied field of 100 Oe. Figures (4), shows the full *M* x *H* hysteresis loops measured at 300 and 2 K in an applied magnetic field up to 4T. At low temperature (2 K), the samples exhibit hysteretic behavior (Fig. 4b) that is typical for ferromagnetic features with finite coercive fields $\:{H}_{c}^{2K}$ = 408, 269 and 295 Oe and remanance $\:{M}_{r}^{2\:K}$= 7, 6.6 and 17.5 emu/g for 0, 5 and 9% Mn-doped IONPs, respectively. However, the hysteresis loops measured at 300 K imply super-paramagnetic features with zero remnant magnetization and coercivity. The transition from a non-hysteretic behavior at room temperature to a hysteretic one at 2 K suggests that the nanoparticles undergo a blocking process upon cooling, which is consistent with typical superparamagnetic behavior. In bulk samples, where the particle size is larger than the width of the domain wall, magnetization behavior is primarily governed by the domain wall motion. When the applied field varies, domain walls motion can become pinned at grain boundaries leading to remnant magnetization and coercivity due to the additional energy required for continued domain wall motion. Pinning of the domain walls is one of the main contributions to the coercivity and remnant magnetization of the magnetic materials^44^. As long as the particle size D remains larger than a critical value D_cr_, reducing the particle size is expected to create more pinning centers resulting in an increase of H_C_ (H_C_ α D^−1^). However, when the particle size is reduced below D_cr_ (the single domain size), H_C_ decreases rapidly (H_C_ α D^6^) i.e., D_cr_ represents the maximum particle size at which magnetic moments can reverse coherently.

It is predicted that D_cr_ is represented by the relation D_cr_ = πS[J/Ka_o_]^1/2^, where, *S*,* J*, *K* and a_o_ are the spin moment per atom, the exchange energy density, the magnetocrystalline anisotropy and the lattice parameters, respectively^45^. It has been reported by Li et al.^46^ that correlating particle size with both magnetic coercivity and crystallite size yields a critical size of approximately 76 nm for iron oxide nanospheres. The particle size extracted from HRTEM investigation confirm that D < D_cr_ is realized for our samples which is consistent with the observed zero coercivity and remnant magnetization.

The superparamagnetic behavior of our samples is confirmed by the nonsaturation of the M x H loops even under relatively high external magnetic fields up to 4 T. This observation is consistent with the fact that the nanoparticles (NPs) under study are typical single domain magnets, making them potential candidates for using as magnetic resonance imaging contrast agents and therapeutic agents in cancer treatment. A quantitative analysis of the hysteresis loops at 300 and 2 K yields magnetization at values at the maximum applied magnetic field 4 T of $\:{\text{M}}_{4\:\text{T}}^{300\:\text{K}}$= 19.7, 21.4 and 43 emu/g and $\:{\text{M}}_{4\:\text{T}}^{2\:\text{K}}$ = 21.9, 24.1 and 49.7 emu/g for 0, 5 and 9% Mn-doped IONPs samples, respectively. $\:{\text{M}}_{4\:\text{T}}^{300\:\text{K}}$ Values are much lower than the saturation magnetization of bulk iron oxide (74.3 emu/g)^47^. This reduced magnetization can be attributed to the spin disorder in the surface layer or existence of other iron oxide phases (other than magnetite) that are paramagnetic or have weak ferromagnetic ordering.

The thickness of the disordered spin layer (t) relative to the particle size (D) can be estimated using the following relation^48,49^:

1 $$\:{\text{M}}_{\text{s}}={\text{M}}_{\text{s}}^{\text{b}\text{u}\text{l}\text{k}}(1-6\text{t}/\text{D})$$

Taking $\:{\text{M}}_{4\:\text{T}}^{300\:\text{K}}$ for our samples as approximate values for the saturation magnetization (M_s_) in Eq. 1, disordered spin layer thickness of ~ 1.2, 1.1 and 0.94 nm are estimated for 0, 5 and 9% Mn-doped IONPs, respectively. Existence of disordered spin layer coincides with the deformed layers observed from the HRTEM images. The applied field dependency of the magnetization in our superparamagnetic NPs is poorly represented by the simple Langevin function. However, it fits well with a modified Langevin function (Eq. 2) that accounts for paramagnetic surface contributions caused by the disordered spin layer^50,51^:

2 $$\:\text{M}\left(\text{H}\right)={\text{M}}_{\text{s}}\left[\text{c}\text{o}\text{t}\text{h}\left(\frac{{{\upmu\:}}_{\text{p}}\text{H}}{{\text{k}}_{\text{B}}\text{T}}\right)-\frac{{\text{k}}_{\text{B}}\text{T}}{{{\upmu\:}}_{\text{p}}\text{H}}\right]+{{\upchi\:}}_{\text{a}}\text{H}$$

where µ_p_ is the magnetic moment per particle given by $\:{{\upmu\:}}_{\text{p}}={\text{M}}_{\text{s}}{\uppi\:}{\text{D}}_{\text{m}\text{a}\text{g}}^{3}/6$ emu, k_B_ is the Boltzmann constant, D_mag_ is the particle size, χ_a_ is the high field susceptibility attributed to the paramagnetic contribution caused by the disordered spin layer or any other contributions caused by nonmagnetic derivatives in the sample. The values of saturation magnetization ($\:{\text{M}}_{\text{s}}^{\text{l}\text{a}\text{n}\text{g}}$), magnetic moment (*µ*_*p*_), particle size D_mag_ and paramagnetic contribution χ_a_ extracted from the optimal fitting to Eq. (2) are tabulated in Table 2. The calculated mean particle size D_mag_ values are very close to the average particle size (D_TEM_) determined from the TEM results. The paramagnetic contribution of susceptibility χ_a_ of the undoped sample has a value of 2.75 × 10^−7^ emu/Oe which is comparable with that reported by Nagesha et al. for Fe_3_O_4_ NPs (average particle size D_TEM_=10 nm) synthesized using high temperature thermal decomposition method^50^. However, the value increases significantly and reaches 14 × 10^−7^ and 20 × 10^−7^ emu/Oe by increasing the Mn doping level to 5% and 9%, respectively. This increase in χ_a_ value indicates the role of Mn doping for increasing the paramagnetic contribution due to the associated deterioration in crystallinity, increase in the disordering and formation of nonmagnetic impurities.

**Table 2:** Magnetic parameters of 0, 5 and 9% Mn-doped iron oxide NPs.

Sample	$\:{\varvec{M}}_{4\:\varvec{T}}^{300\:\varvec{K}}$ (emu/g)	$\:{\varvec{M}}_{\varvec{s}}^{\varvec{l}\varvec{a}\varvec{n}\varvec{g}}$ (emu/g)	*µ*_p_ × 10^−24^ (emu)	D_mag_ (nm)	K_eff_×10^4^ (J/m^3^)	χ_a_ x 10^−7^ (emu/Oe)
0%	19.7	19.38	8.79	9.53	23.60	2.75
5%	21.4	21.1	12.50	10.42	9.23	14
9%	43	42.5	9.19	7.45	4.56	20

**Fig. 5:**
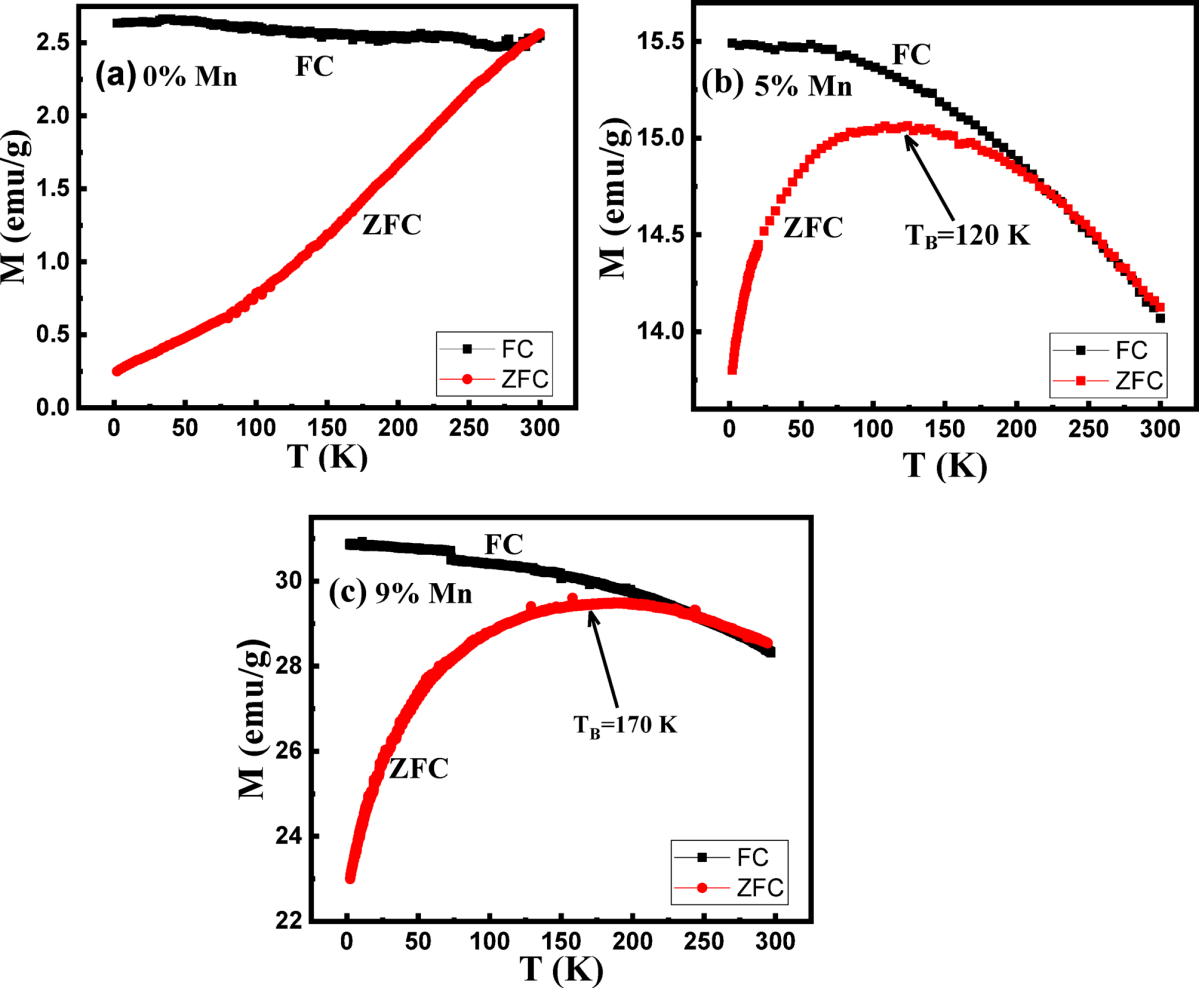
Zero-field-cooled (ZFC) and field-cooled (FC) magnetization plots of (**a**) 0%, (**b**) 5% and (**c**) 9% Mn-doped IONPs.

Figure (5) depicts the temperature dependence of the magnetization (M×T plots) of the NPs. Here, the materials were initially cooled in the ‘‘zero-field-cooled’’ (ZFC) regime to 2 K, after which a magnetic field 100 Oe was applied. In this a ZFC regime, magnetization was measured upon heating up to 300 K. Subsequently, magnetization was measured in the field-cooled (FC) regime while cooling back down to 2 K under the same field (100 Oe). As seen in Fig. (5a), the ZFC magnetization of the un-doped NPs increases sharply upon heating and exhibits a tendency to decline around 300 K. However, the ZFC curve remains lower than the FC curve within the temperature range 2 to 285 K indicating the presence of ferromagnetism up to at least 285 K. The overlapping between ZFC and FC curves that begins at 285 K due to the small particle size, super-paramagnetic behavior is expected at T > 285 K. Consequently, a blocking temperature T_B_ > 285 K is anticipated for the un-doped sample. Assuming a spherical shape for the magnetic nanoparticle, T_B_ can be estimated using the following relation:

3 $$\text{T}_{\text{B}}=\text{K}_{\text{eff}}\text{V/25k}_{\text{B}}$$

where K_eff_ and V are the effective anisotropy constant and the volume of the magnetic particle, respectively^36^. K_eff_ accounts for contributions from various anisotropy including surface, magnetocrystalline, shape and stress anisotropies along with additional contribution from dipolar and/or exchange interactions among the NPs. Based on our observation of T_B_ > 285 K and the value of D_TEM_, the effective anisotropy constant was roughly estimated to be 2.3 × 10^5^ J/m^3^ for the un-doped NPs. This anisotropy constant is higher by one order of magnitude as compared to the bulk values for magnetite (K_eff_ =1.35 × 10^4^ J/m^3^) and 2 orders of magnitude as compared to the bulk values of magnitude for hematite (K_eff_ =0.4 × 10^4^ J/m^3^). The value is close to that reported by Gulati and Jain^52^ (1.45 × 10^5^ J/m^3^) for IONPs with particle size between 30 and 40 nm synthesized by co-precipitation method. However, it is higher than the value 2.5 × 10^4^ J/m^3^ reported by Bianco et al.^18^ for 6.8 nm NPs synthesized from decomposition of organometallic precursors. In general, surface anisotropy plays a key role in tuning the total magnetic anisotropy of the NPs. As particle size increases, total magnetic anisotropy decreases due to the enhancement of the surface anisotropy. Thus, both the composition (existence of one or more other iron oxide phase/s such as hematite, maghemite and Wüstite as secondary phase) and size of NPs govern the total magnetic anisotropy^53–55^.

For the Mn-doped samples, the ZFC plots exhibit broad maxima (see Fig. 5b &c) indicating a wide distribution of blocking temperatures rather than a well-defined value. This behavior suggests a broad particle sizes distribution leading to a wide range of energy barriers. The blocking temperature, extracted from the maxima of the ZFC plots was found to be 120 K and 170 K for the 5 and 9% Mn-doped samples, respectively. The magnetic anisotropy (K_eff_) was estimated using Eq. (3) and is presented in Table (2). The data indicate that K_eff_ decreases with increasing the Mn doping reaching 4.56 × 10^4^ J/m^3^ for the 9% Mn-doped sample. As an example of the effect of Mn doping on the magnetic anisotropy, Guati and Jain^52^ reported that increasing the Mn doping level to 20% reduced the magnetic anisotropy of iron oxide by 66%. Further experimental studies are necessary to fully understand this effect in detail. ZFC and FC plots of 0, 5 and 9% Mn-doped samples overlap at irreversibility temperature T_irr_ = 285, 245 and 220 K, respectively. T_irr_ is related to the blocking of the largest particles. T_B_ and T_irr_ coincide for an ideal superparamagnetic material with narrow particle distribution thus their difference provides a good monitor for the authentic particle size distribution^36^. Therefore, the observed irreversibility of ZFC and FC plots to T_irr_ higher than T_B_ indicates the relatively wide particle size distribution of our samples.

**Fig. 6:**
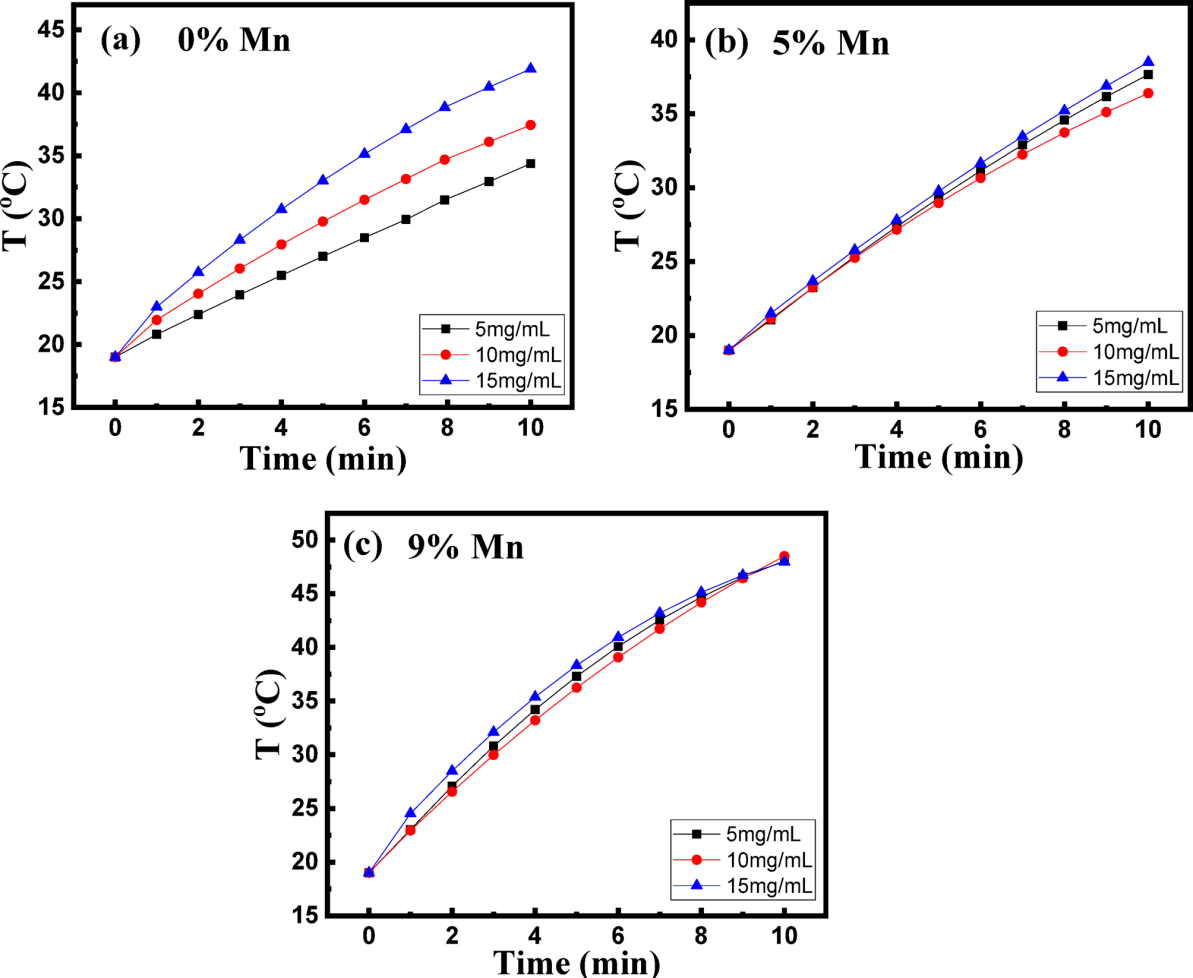
Temperature (^°^C) vs. time (min) upon application of AC magnetic fields for (**a**) 0, (**b**) 5 and (**c**) 9% Mn-doped IONPs with different concentrations of NPs suspension.

The magnetic heating properties under applied AC magnetic fields were investigated to evaluate the feasibility of the synthesized NPs for magnetic hyperthermia. The temperature released by the magnetic NPs versus time T(t) was measured under applied AC magnetic field 6.3 kA/m with a fixed frequency 300 kHz. The experiments were conducted on nanoparticle suspensions with concentrations of 5, 10, and 15 mg/mL, with each suspension being sonicated for 2 min to improve nanoparticle dispersibility. Figure 6 shows the time-dependent calorimetric plots for the suspensions at different NPs concentrations. All suspensions exhibit significant heating response at 5 mg/ml. The heating rate of the un-doped and 5% Mn-doped NPs increases from 1.55 to 1.86 to 2.29 and 1.95 °C/s, respectively with increasing the suspension concentration from 5 to 15 mg/ml. However, the heating rate of the 9% Mn-doped NPs shows approximately the same value ~ 2.9 for all the suspensions with different NPs concentrations. This may be attributed to agglomeration of some particles to larger magnetic agglomerates in the suspension that occurs at higher concentrations particularly because of the 9% Mn-doped NPs shows the highest magnetic ordering.

The actual heating efficacy of magnetic particles in AC magnetic fields is quantitatively represented in terms of specific absorption rate (SAR)^51^:

4 $$\:SAR=C\frac{dT}{dt}\frac{1}{{m}_{act}}$$

where C, dT/dt and m_act_ are the specific heat of water, initial slope of the temperature raise and mass of the active magnetic material (mass of magnetic particles/total suspension mass). The SAR represents the heating ability of magnetic particles and, thus, the feasibility of a material for using as a hyperthermia agent. To minimize the amount of the NPs applied for hyperthermia, the SAR value should be as high as possible. As seen in Table (3), SAR value, at fixed NPs concentration, increases with increasing the Mn content. Interestingly, for un-doped and Mn-doped samples, the values of SAR are higher for suspensions of lower NPs concentrations as seen in Table 3. This may be attributed to that at higher particle concentration, the particles agglomerate in larger entities and the magnetic heating efficiency decreases due to stronger dipole–dipole interactions^56^. Similar results have been reported by Islam et al.^57^ for Mn-doped iron oxide NPs synthesized by chemical co-precipitation method.

**Table 3:** Specific absorption rate (SAR) of the 0, 5, 9% Mn-doped ions with different concentrations of NPs suspension.

Sample	Concentration mg/mL	SAR (W/g)
0% Mn	5	1210.5
	10	649.1
	15	498.2
5% Mn	5	1506.4
	10	685.5
	15	515.5
9% Mn	5	2449.4
	10	1224.7
	15	769.1

Numerous studies have reported the hyperthermia performance of IONPs with different size, geometry, and anisotropy under different AC magnetic fields^58^. Spherical Fe_3_O_4_ NPs, with sizes ranging from 30 to 50 nm, synthesized by Nemati et al.^59^ exhibit SAR value of 650 W/g at 800 Oe and 310 kHz. Iron oxide nanorods, nanocubes, and nanospheres with approximately near sizes were synthesized by Das et al. and exhibited SAR values of 862 W/g, 314 W/g and 140 W/g, respectively^60^. Bae et al.^61^ synthesized chitosan oligosaccharide-coated 30 nm-sized Fe_3_O_4_ NPs which exhibited SAR values of 2614 W/g. Generally, it can be concluded that the sizes and geometries are the main factors that can tune the magnetic heating capability of iron oxide.

## Materials and methods

### Materials

Fresh sugarcane juice was obtained from a local vendor and filtered through a Whatmann filter paper no. 41. Fe_2_(No)_3_.6H_2_o (LOBA chemie 99.4%), MnSO_4_.H_2_O (LOBA chemie), were used as precursors in the synthesis process. Sodium hydroxide [NaOH] (Sigma Aldrish 99.99%) was used to adjust the pH. Deionized distilled water was selected as the solvent for this preparation process. All the chemicals and solvents used were of analytical reagent grade.

### Biosynthesis of iron oxide NPs with sugarcane juice

**Fig. 7:**
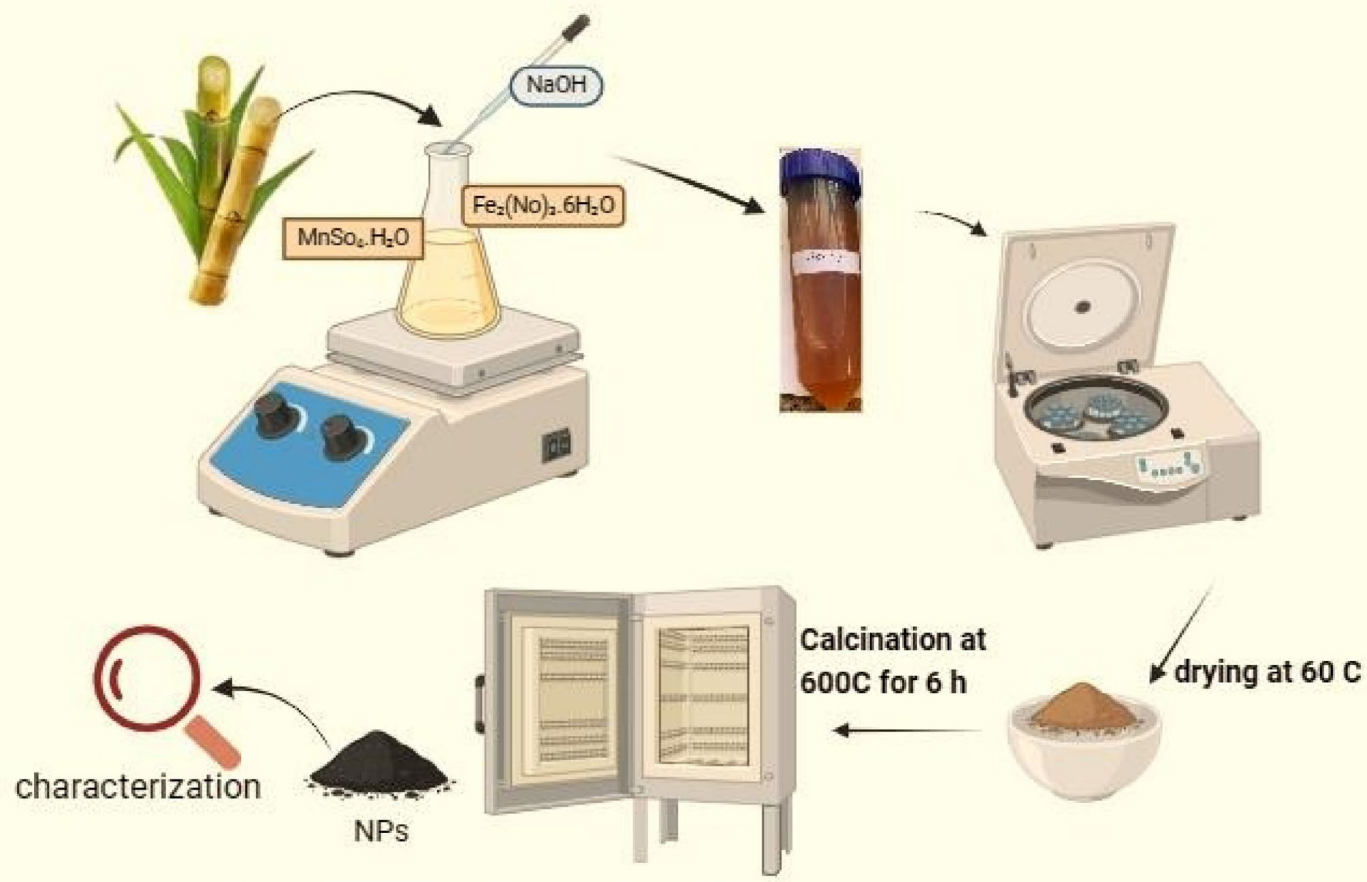
Schematic diagram of green synthesis of IONPs and Mn-doped IONPs by ScJ.

To synthesize iron oxide nanoparticles (IONPs) 30 ml of filtered juice was added dropwise to 100 mM Fe_2_(NO)_3_.6H_2_O (LOBA chemie 99.4%) in a 500 ml flask and stirred at 50 ˚C for one hour. The color of the solution changed from light brown to dark brown indicating the formation of iron oxide nanoparticles (IONPs). To prepare manganese doped iron oxide nanoparticles, MnSO₄.H₂O was added at 5% and 9% atomic ratios to the iron salt before reduction with 30 ml sugarcane juice (Fig. 7). The pH of the reaction was maintained at approximately 10 by adding NaOH with vigorous stirring. To remove possible residual ions and organic impurities, the precipitate obtained was centrifuged, thoroughly washed with distilled water and dried in a hot air oven at 60 ˚C for 24 h. The cleaned precipitate was then sintered in a muffle furnace at 600 ˚C for 6 h. The Final powder was used for nanoparticle characterizations, magnetic properties analysis, and hyperthermia applications.

### Physical characterization

#### X-Ray diffraction (XRD)

X-ray diffraction (XRD) measurements were carried out using a D8-Advance, Bruker diffractometer with Cu-K_α_ radiation (λ = 1.54 Å), over the 2θ collection range of 20–80^°^ using an angle step of 0.02^°^and step time of 0.6 s. The patterns were recorded at 40 kV and 30 mA. Si low background sample holder was used. The average crystallite size (D_XRD_) of iron oxide NPs can be estimated from the XRD patterns using the Debye–Scherrer formula^62^:

5 $$\:\:\:\:\:D\text{XRD}=\frac{K\:\lambda\:}{\beta\:cos\theta\:}\:\:$$

where D_XRD_ is the crystallite size of NPs (in nm), K is a constant related to the crystallite shape (K = 0.9), λ is the wavelength of Cu-K_α_radiation, β is the full width at half maximum (FWHM) of the most predominant peaks (in radians) and θ is the diffraction angle (in radians). Also, the lattice parameters and cell volume for hexagonal structure of pure and Mn-doped iron oxide were determined using the following Eqs^62,63^.:

6 $$\:\frac{1}{{d}^{2}}=\frac{4}{3}\left(\frac{{h}^{2}+hk+{k}^{2}}{{a}^{2}}\right)+\frac{{l}^{2}}{{c}^{2}}$$

7 $$\:V=0.866{a}^{2}c$$

Additionally, the micro strain (η) and dislocation density $\:\left(\delta\:\right)$for biosynthesis samples were calculated from XRD data to indicate the amount of deformations in lattice structure using Eq^64^.:

8 $$\:\eta\:=\frac{{\beta\:}_{hkl}{cos}\theta\:}{4}$$

9 $$\:\delta\:=\frac{1}{{D}^{2}}$$

#### Fourier transform infra-red (FTIR) spectra

Fourier Transform Infra-Red spectra (Alpha FTIR Bruker), in Sohag University, Egypt, for SCJ, iron oxide, and Mn-doped iron oxide powders were recorded to investigate the chemical functional groups of the surface.

#### Transmission electron microscopy (TEM)

TEM analysis was done using high-resolution transmission electron microscopy (HR-TEM) (JEOL, JEM-2100). For the analysis, samples were dispersed in ethanol, sonicated and sprayed on a carbon-coated copper grid and then allowed to air drying, finally, Gatan SOLARUS 950 was used before observation.

### Magnetic properties of ionps

Magnetic measurements were carried out using a Quantum Design Physical Property Measurement System (PPMS) instrument. The saturation magnetization was measured as a function of the applied magnetic field at room temperature and 2 K. Also, temperature dependence of magnetization was measured in zero field- and field-cooling regimes at a fixed applied magnetic field of 100 Oe and within the temperature range from 2 to 300 K.

### Heating efficiency of ionps

The heating efficiency of the un-doped and Mn-doped iron oxide NPs were tested using HT equipment. The magnetic induction heating device includes a Zero Voltage Switch (ZVS) that converts DC to AMF and an isolated sample container confined by a solenoid induction copper coil. The coil was built of 7-turn coil copper tubing with 3 cm radius, and it was water-cooled^65^. The tests were carried out at fixed frequency of 300 kHz, a current generator of (14.6 A DC), and magnetic field strength of (6.3 kA/m). The heating rate was measured as a function in time. The specific absorption rate SAR (W/g) was evaluated using the initial slope of the temperature change with time (dT/dt).

## Conclusion

In this study, Mn-doped IONPs were successfully synthesized using sugarcane juice as a green synthesis route. XRD analysis confirmed that the product consisted of Fe_3_O_4_ cubic spinel structure. HRTEM revealed semi-spherical nanoparticles with average particle size 9.3, 9.5, and 13.5 nm for 0, 5, and 9% Mn-doped iron oxide, respectively. The magnetic characterization showed that the nanoparticles exhibited a ferromagnetic hysteresis loop at 2 K and superparamagnetic behavior at 300 K. The obtained magnetic parameters for the superparamagnetic behavior fit well with modified Langevin function. The blocking temperature was extracted from the maxima of the ZFC plots to be ~ 285, 120, and 170 K for 0, 5 and 9% Mn-doped samples, respectively. The values of Specific Absorption Rate (SAR) values increased with Mn-doping, demonstrating improved heating efficiency in AC magnetic fields. However, suspensions of lower NPs concentrations recorded better SAR values due to stronger dipole–dipole interactions at high concentration of magnetic NPs. The synthesized Mn-doped IONPs show great promise for biomedical applications, particularly in magnetic hyperthermia-mediated drug delivery and cancer therapy. This study further highlights the potential of green synthesis methods in developing functional nanomaterials for advanced medical applications.

## Data Availability

The authors declare that the data supporting the findings of this study are available within the paper.
